# Case Report: Feasibility of clinic-to-home multichannel FES-assisted gait rehabilitation in secondary progressive multiple sclerosis

**DOI:** 10.3389/fresc.2026.1939617

**Published:** 2026-10-22

**Authors:** Michael Leal, Amine Metani, Perrine Séguin, Maël Descollonges

**Affiliations:** Research & Development Department, Kurage, Villeurbanne, France

**Keywords:** case report, FES-assisted exercise, functional electrical stimulation, gait rehabilitation, multiple sclerosis, quantified gait analysis

## Abstract

**Background:**

Functional electrical stimulation (FES) is used as an adjunct to gait rehabilitation in people with multiple sclerosis (PwMS), but most approaches target a single muscle group. This case report describes the feasibility, tolerability, and observed functional changes associated with a novel multichannel FES (NeuroSkin®) system, to our knowledge the first report of its use across clinic-to-home phases in PwMS, in a 61-year-old woman with secondary progressive multiple sclerosis (EDSS 6) with gait and balance impairment requiring a cane for community ambulation.

**Methods:**

This retrospective case report analyzed prospectively collected routine-care data covering an 18-week multimodal FES-assisted rehabilitation program comprising 58 sessions of 60–80 min each: a 7-week clinic-based phase (23 sessions), a 6-week supervised home-based phase (20 sessions), and a 5-week unsupervised home-based phase (15 sessions). Sessions included motor imagery, sit-to-stand training, FES-assisted cycling, and overground gait training. Multichannel stimulation was delivered in a gait-phase-specific manner (40 Hz; pulse widths 200–400 µs) according to muscle group. Functional outcomes were assessed at baseline (PRE), after the clinic-based (MID1), supervised home-based (MID2), and unsupervised home-based (POST) phases, using the Functional Reach Test (FRT), 10-m Walk Test, and Timed Up and Go (TUG); data were analyzed descriptively.

**Results:**

No serious adverse events occurred; the participant experienced transient skin redness, and device deficiencies in 3 of 58 sessions, all resolved without affecting completion. Walking speed on the 10-m Walk Test increased from 0.53 to 1.04 m·s⁻^1^ (+96%), and TUG time decreased from 17 to 11 s (−35%); both changes exceeded published minimal detectable change thresholds for these measures in MS. FRT improved from 32 cm at PRE to 40 cm at MID1 (+25%) and 42 cm at MID2 (+31%) but returned to the baseline value (32 cm) at POST. Quantified gait analysis showed higher walking speed and step length during the supervised phases than the unsupervised phase. The participant completed all 58 sessions.

**Conclusions:**

Despite inherent limitations of a single retrospective case report, these findings indicate that a multichannel FES-assisted rehabilitation program is feasible and well tolerated in a person with advanced SPMS across care settings; controlled studies are needed to establish efficacy.

## Introduction

Multiple sclerosis (MS) is a chronic autoimmune condition characterized by the loss of myelin and neurodegeneration in the central nervous system (CNS) (1). Globally, it is the leading cause of neurological disability among young adults (2, 3). Most people with MS (PwMS) experience walking impairments that are clinically significant and considered among the most disabling consequences of the disease (3–5). PwMS typically demonstrate altered spatiotemporal gait parameters, including reduced walking speed, shorter step length, increased step duration, and prolonged double support phase (6). Thus, physical exercise and gait rehabilitation have been recommended as pivotal rehabilitation approaches for managing functional capabilities and symptomatic outcomes in PwMS (7). However, physical exercise alone can sometimes be challenging due to the severity of the functional impairments caused by the disease. To counteract this, functional electrical stimulation (FES) has been reported as a good alternative for neurological disorders, in particular for PwMS (3). In short, FES induces muscle contractions to initiate or assist movement and, when synchronised with walking cycles, can support gait function. Most studies using FES in PwMS have focused on FES-assisted cycling or neuroprostheses targeting a single muscle group. Although such studies have investigated the use of multichannel functional electrical stimulation for gait rehabilitation in stroke (8) and spinal cord injury (SCI) populations (9–11), only a limited number of studies have explored its application in people with multiple sclerosis (PwMS). While home-based FES rehabilitation programs have already been investigated with positive effects, notably in post-stroke or in MS people (12), to the best of our knowledge, the combination of a center-based phase followed by a home-based FES program, especially using a novel device combining artificial intelligence and FES, has never been specifically evaluated in PwMS. In this context, although only a few devices integrating sensors for FES are currently available for MS rehabilitation, a medical device named NeuroSkin® (Kurage, Villeurbanne, France) has recently been developed. NeuroSkin® combines a multichannel FES with a wearable gait analysis system incorporating embedded inertial measurement units and foot pressure sensors (8, 13). From this perspective, the present case report was conceived as a proof-of-concept study. This retrospective analysis aimed to examine pre-existing data to evaluate the impact of this innovative multichannel FES device on spatiotemporal gait parameters in a single individual diagnosed with MS, across both clinical, center-based, and home-based rehabilitation settings.

### Case description

The participant was a 61-year-old woman (weight: 63 kg; height: 158 cm) diagnosed with secondary progressive multiple sclerosis (SPMS) in 2001. At the time of the intervention, she was classified as having an Expanded Disability Status Scale (EDSS) score of 6, indicating the need for a unilateral walking aid (e.g., cane or crutch) to ambulate approximately 100 meters, with or without rest. The diagnosis of SPMS and the EDSS score of 6 were established by the participant's referring neurologist prior to her enrolment in this program. This participant was selected for retrospective review and publication because she had already been equipped with a personal NeuroSkin® device as part of her routine clinical care, and because she completed the entirety of the planned 18-week, three-phase program, providing a rare, complete dataset spanning clinic-based, supervised home-based, and unsupervised home-based use. Her primary reported concern was progressively worsening gait and balance. The participant presented with mild weakness of the right lower limb, with no impairment of the upper limbs. The participant presented a combination of distal deficits (reduced foot clearance during swing), proximal impairments (reduced propulsion and lower-limb weakness), impaired balance, and fatigue-related gait deterioration. A unilateral cane was required for community ambulation. The clinical presentation did not meet the criteria for hemiplegia or paraplegia but was consistent with monoparesis. From a cognitive perspective, the participant had no known cognitive impairment and no history of depression. Regarding spasticity, bilateral calf muscles exhibited mild spasticity, with a Modified Ashworth Scale (MAS) score of 1. This spasticity occurred predominantly at night and manifested as muscle spasms disrupting sleep. The participant was receiving ongoing pharmacological treatment with oral baclofen and was also equipped with a multichannel functional electrical stimulation device (NeuroSkin®) as part of her rehabilitation management. Prior to enrolment in this program, the participant had received ongoing physiotherapy follow-up for her multiple sclerosis, but had no prior exposure to functional electrical stimulation, with the outcomes of this prior therapy not documented in the retrospective record. The participant provided written informed consent for the use of her clinical data collected during the rehabilitation period for research purposes.

### Diagnostic assessment

All functional outcome measures were assessed at four time points: at baseline, before the initiation of the rehabilitation program (PRE); after the 7-week clinic-based phase (MID1; 23 sessions); after the supervised home-based phase (MID2; 20 sessions); and after completion of the 18-week FES-based intervention, corresponding to the end of the unsupervised home-based phase (POST; 15 sessions) (Figure 1). The PRE, MID1, MID2, and POST assessments correspond to routine clinical follow-up evaluations embedded within the standard physiotherapy care pathway, rather than predefined research timepoints. This study is a retrospective analysis of prospectively collected clinical data from routine care. All clinical assessments were conducted under identical conditions by the same trained examiner to ensure consistency and reduce inter-rater variability. Functional balance and postural control were evaluated using the functional reach test (FRT) (14). This test assesses the maximal distance a participant can voluntarily reach forward beyond arm's length while maintaining a fixed base of support in standing. Walking speed was assessed using the 10-m Walk Test. The participant was instructed to walk at a comfortable self-selected speed over a 10-meter distance, and the time required to complete the distance was recorded to calculate gait speed. Global functional mobility, integrating transfers, walking, turning, and sitting, was assessed using the Timed Up and Go test (TUG). The participant was asked to stand up from a seated position, walk three meters, turn around, return to the chair, and sit down as quickly and safely as possible. The total time to complete the task was recorded. Additionally, quantitative gait analysis data were recorded concurrently during each FES-assisted walking session. Step length and walking speed data were collected during each FES-assisted walking phase.

**Figure 1:**
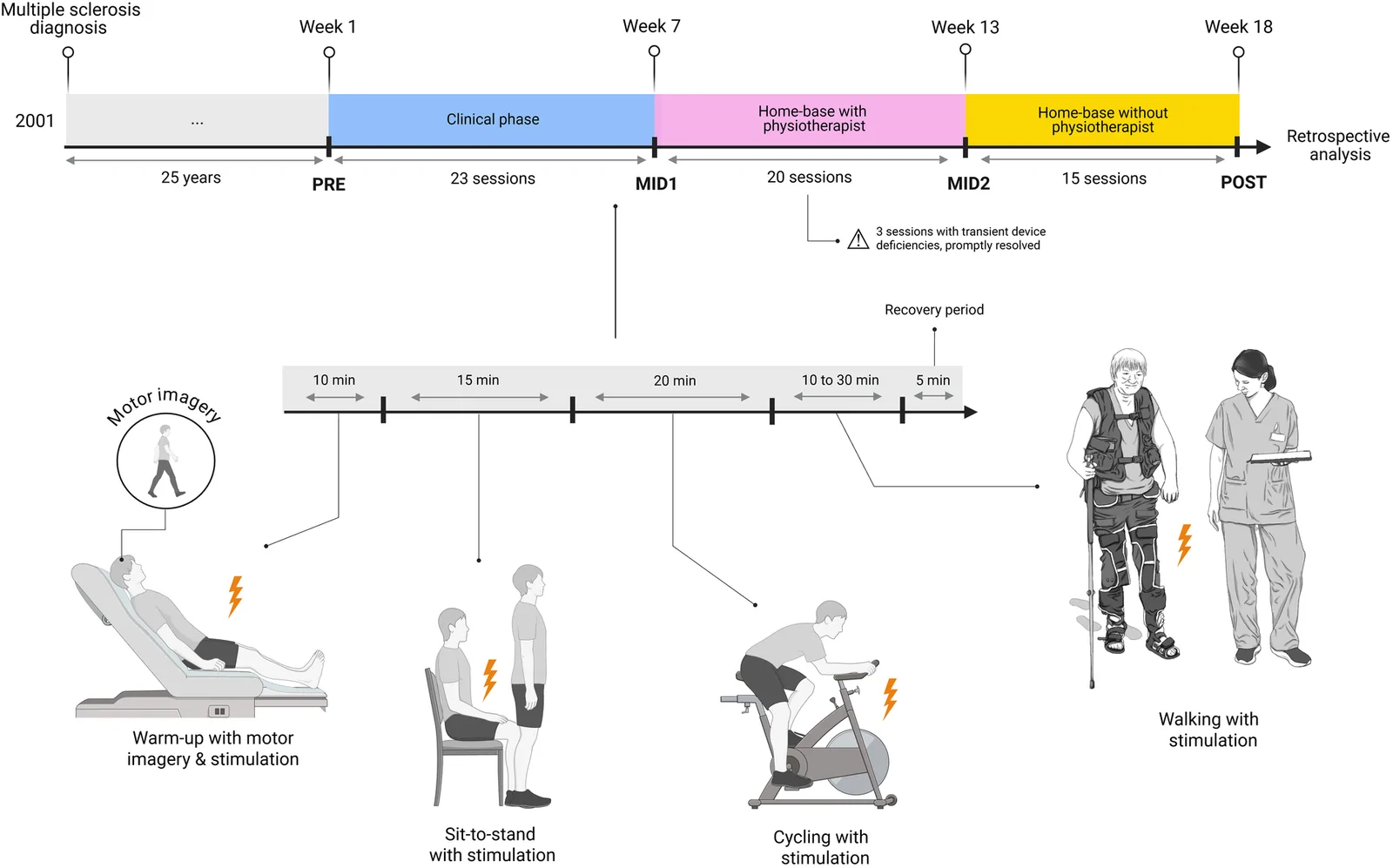
Clinical and intervention timeline. The timeline shows the retrospective analysis window relative to the participant's SPMS diagnosis (2001), the three intervention phases (clinic-based, weeks 1–7, 23 sessions; home-based with physiotherapist supervision, weeks 8–13, 20 sessions; home-based without supervision, weeks 14–18, 15 sessions), and the four assessment timepoints (PRE, MID1, MID2, POST). The lower panel illustrates the structure of a typical session. Human illustrations are original drawings with invented facial features that do not depict the participant's actual likeness.

### Therapeutic intervention

#### Functional electrical stimulation device

The NeuroSkin® system (Kurage, France) is a CE-marked, wearable lower-limb FES system designed to support gait rehabilitation. It consists of a fitted textile garment integrating dry electrodes positioned over six major muscle groups on each leg (Gluteus Maximus, Quadriceps, Hamstrings, Tibialis Anterior, Fibularis longus, and Triceps surae) (8, 11, 13). The garment is complemented by an array of inertial sensors (seven IMUs placed on the pelvis, thighs, shanks, and feet) and by instrumented insoles embedding eight plantar pressure sensors per foot. A lightweight processing unit worn on the upper body runs a real-time gait-phase detector, while a MotiMove stimulator (3F-Fit Fabricando Faber by Kurage, Serbia) delivers patterned multichannel FES (8, 13). Stimulation parameters and safety limits are configured through a dedicated tablet application (8). These allow the physiotherapist to adjust frequency, pulse width, and muscle-specific timing. In addition to being configured before each session, all stimulation parameters, intensity, pulse width, frequency, and muscle-specific gait-phase timing, could be adjusted in real time by the physiotherapist through the tablet application's expert-level settings while the participant was actively exercising or walking, allowing on-the-fly modulation according to the participant's comfort, fatigue, or performance. Typical stimulation values ranged from 200 to 400 µs in pulse width, depending on the muscle group, delivered at 40 Hz. Pulse widths were set to 400 µs for the quadriceps, gluteals, and hamstrings, 250 µs for the triceps surae, and 200 µs for the tibialis anterior and fibularis longus muscles. Further, to improve electrode-skin contact, a moisturizing lotion was applied before donning the garment (15). Stimulation intensity is independently adjustable per channel/muscle group through the device application, in addition to frequency and pulse width. Device donning took approximately 5–8 min and doffing approximately 2 min. Donning time was comparable when performed by the participant herself during the unsupervised phase, though it could vary depending on her day-to-day fatigue level; this variability was not systematically recorded. Initial intensities and gait calibration took ∼10 min; per-session recalibration, ∼30 s. Battery life is ∼4 h per charge; the system requires Wi-Fi.

Before the training program, the participant completed a calibration and personalization procedure. Calibration determined maximum tolerable stimulation levels for each muscle confirmed by the physiotherapist as producing a visible, non-painful motor response. A brief ∼10-step recalibration was additionally performed at the start of every subsequent session throughout all three phases, to account for day-to-day variability. Personalization consisted of a short walking trial without stimulation, which allowed the system to fine-tune the general gait model, initially developed from recordings of healthy and post-stroke individuals to the participant's individual gait characteristics.

#### Embedded quantified gait analysis (QGA)

In addition to delivering functional electrical stimulation, the NeuroSkin® system enables embedded quantified gait analysis (QGA) during FES sessions. The combination of seven IMUs and instrumented insoles provides continuous, real-time capture of lower-limb kinematics and plantar pressure during walking. QGA extracts spatiotemporal parameters such as step length and walking speed.

#### Rehabilitation sessions

The participant completed a total of 18 weeks of rehabilitation exclusively based on functional electrical stimulation (FES)-assisted exercise with the NeuroSkin device® (8, 11)(see description below), comprising 58 sessions in total. The intervention was divided into three consecutive phases: (1) an initial clinic-based phase during the first seven weeks; (2) a home-based phase with physiotherapist supervision from week 8 to week 13; and (3) a final home-based phase without physiotherapist supervision from week 14 to week 18. The participant performed three to five sessions per week depending on fatigue and scheduling constraints. During the supervised phases, all sessions were conducted under the guidance of a physiotherapist trained in the use of the FES system.

Each session lasted approximately 60–80 min, depending primarily on the duration of the gait-training component, and followed a standardized structure. Sessions systematically began with a 10-minute seated warm-up during which alternating multichannel electrical stimulation was applied to the lower limbs in a walking-like activation pattern at motor threshold. During this phase, the participant was instructed to keep her eyes closed and to perform motor imagery by imagining herself walking. Following the warm-up, the participant performed 15 min of sit-to-stand training assisted by FES. This phase consisted of four sets of ten repetitions, performed with external weights of 2, 4.5, 7, and 9 kg. During this exercise, phase-specific electrical stimulation was applied during both concentric and eccentric phases of the movement. Bilateral stimulation targeted the gluteal and quadriceps muscles, as well as the hamstrings and triceps surae muscles, to support controlled execution of both the ascending and descending phases. The session then continued with 20 min of FES-assisted cycling performed on a recumbent cycle exercise bike (EV100RC, Everlast, USA). This modality was selected due to its ability to reduce the perception of effort (16, 17) while providing repetitive, rhythmic lower-limb movements, muscle hypertrophy (18) cerebro- and cardiovascular stimulation (16, 19–21). The participant was instructed to pedal at a moderate and self-selected cadence. Stimulation intensity was systematically adjusted to the maximal level tolerable by the participant (22). The final active phase of each session consisted initially of up to 10 min of FES-assisted gait training performed indoors, including obstacle negotiation and stair ascent and descent. When feasible, outdoor walking was also included. The duration of the walking phase was adapted according to the participant's day-to-day fatigue level: in some sessions, walking time could be shorter than 10 min, whereas when the participant felt capable, the therapist could extend the gait training period, up to a maximum of 30 min. Each session concluded with a five-minute recovery period in a semi-recumbent position to allow recovery.

### Follow-up and outcomes

#### Intensity of stimulation

Quadriceps: 3–50 mA (mean 26 mA), tibialis anterior: 2–41 mA (mean 17 mA), fibularis longus: 3–33 mA (mean 18 mA), hamstrings: 3–49 mA (mean 25 mA), gluteal muscles: 2–46 mA (mean 24 mA), and triceps surae: 3–47 mA (mean 24 mA). Intensities for each specific type of exercise are presented below in Table 1.

**Table 1:** Mean intensities (mA) by muscle and exercise type. NA: Non-applicable. Values represent means pooling all contributing sessions and both muscle sides together, intended to give an overall order of magnitude of stimulation intensity across the intervention; variability, range, and *n* per cell are not shown.

Mean intensities for each exercise task (mA)—Both sides averaged						
	Quadriceps	Hamstrings	Tibialis anterior	Fibularis longus	Triceps surae	Gluteus
Warm-up	24	23	19	23	23	20
Sit-to-stand	27	21	9	10	27	22
Cycling	28	29	21	NA	24	28
Gait	26	26	20	20	23	25
Overall mean	**26**	**25**	**17**	**18**	**24**	**24**

#### Feasibility, tolerance, and safety

No serious adverse events (defined as any event requiring medical intervention, hospitalization, or study discontinuation) occurred throughout the 18-week study period. The participant exhibited transient skin redness after some training sessions; this was not systematic and might be attributed to exercise-induced changes in skin vascularization. Duration, recurrence, and any treatment of these episodes were not systematically documented beyond visual observation by the treating physiotherapist. Device deficiencies (technical malfunctions not affecting participant safety) occurred in 3 of 58 sessions and were promptly resolved, either immediately or within a few hours, without preventing the participant from completing the sessions.

#### Balance and mobility

Across the four assessment sessions (PRE, MID1, MID2, and POST), the participant demonstrated marked improvements in functional balance and mobility. Functional Reach Test (FRT) performance increased from 32 cm at PRE to 40 cm at MID1 (+25%) and 42 cm at MID2 (+31%), before returning to 32 cm at POST, suggesting initial gains in balance that were not fully maintained. Gait speed on the 10-m Walk Test improved steadily from 0.53 m·s^−1^ at PRE to 0.84 m·s^−1^ at MID1 (+58%), 1.01 m·s^−1^ at MID2 (+91%), and 1.04 m·s^−1^ at POST (+96%). Similarly, Timed Up and Go (TUG) times decreased from 17 s at PRE to 11 s at MID1 (−35%), 10.6 s at MID2 (−38%), and 11 s at POST (−35%), indicating faster functional mobility (Table 2).

**Table 2:** Changes in functional mobility and gait performance across the intervention. Functional Reach Test (FRT, cm), 10-m Walk Test (m·s⁻^1^), and Timed Up and Go (TUG, s). Values represent a single trial per timepoint; no replicate-trial variability is available. For the TUG, a negative change indicates performance improvement.

	PRE	MID1	MID2	POST	*Δ*% change (MID1-PRE)	*Δ*% change (MID2-PRE)	*Δ*% change (POST-PRE)
FRT (cm)	32	40	42	32	+25%	+31%	0%
10-m Walk Test (m·s^−1^)	0.53	0.84	1.01	1.04	+58%	+91%	+96%
TUG (s)	17	11	10.6	11	−35%	−38%	−35%

### Walking speed and step length across the FES-assisted gait phase

Gait speed and step length were plotted chronologically across all 58 sessions to visualize their evolution across care settings (Figures 2A,B). During the clinic-based phase, gait speeds ranged approximately from 25 to 80 cm·s⁻^1^ and step lengths from 20 to 55 cm. During the supervised home-based phase, gait speed (up to >75 cm·s⁻^1^) and step length (up to 60 cm) were higher, with greater session-to-session variability than during the clinic-based phase. During the unsupervised home-based phase, gait speed (50–60 cm·s⁻^1^) and step length (35–45 cm) clustered within a narrower range and were lower than during the supervised phases.

**Figure 2:**
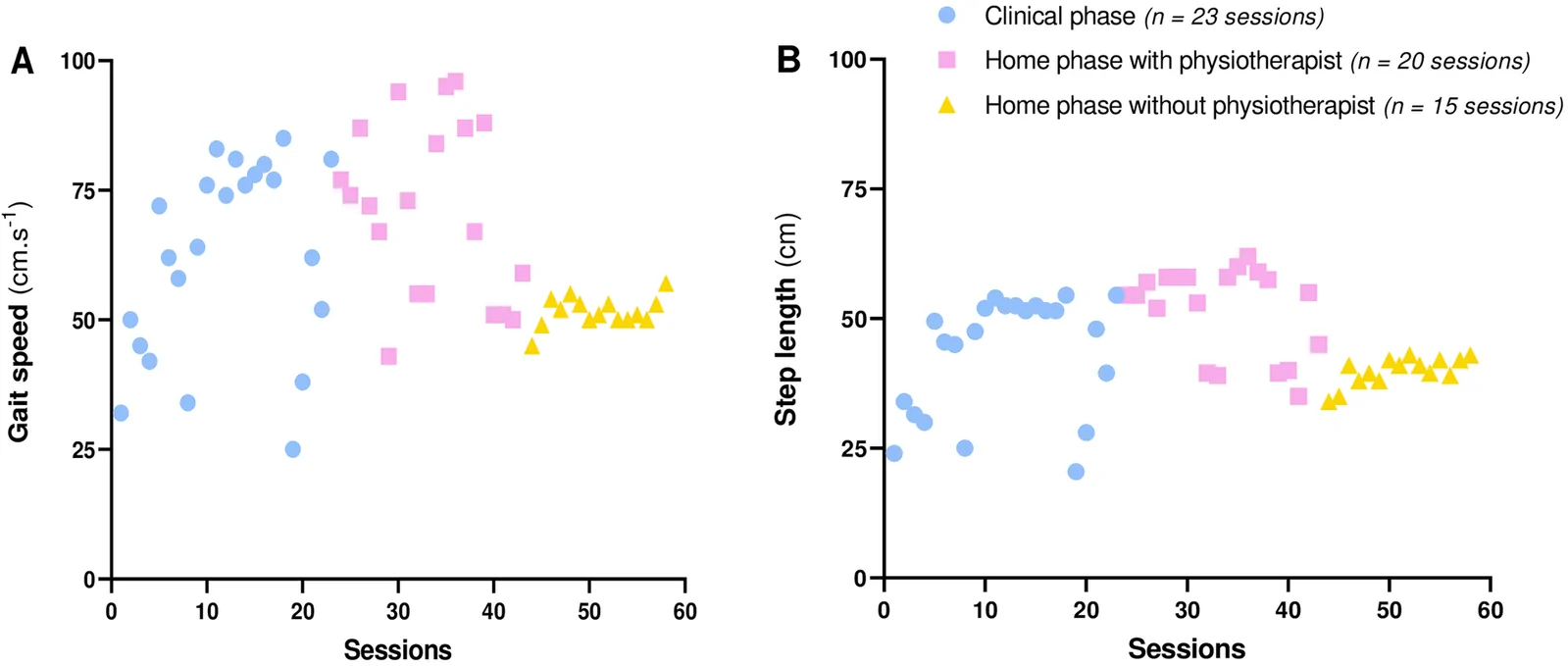
Panels show gait speed **(A)** and step length **(B)** plotted chronologically across all 58 sessions (session number on the *x*-axis), coloured by intervention phase (clinical phase, *n* = 23; home phase with physiotherapist, *n* = 20; home phase without physiotherapist, *n* = 15). Each point represents one session. Data were recorded continuously by the device's embedded sensors during FES-assisted walking (FES active throughout). No sessions had missing or invalid gait data. As these metrics are recorded by the same device that delivers the intervention, and given the absence of independent validation in people with SPMS, they should be interpreted as exploratory observations rather than independently validated efficacy endpoints.

## Discussion

This case report explored the feasibility, tolerance, and preliminary functional effects of a hybrid clinic-to-home, multichannel FES multimodal rehabilitation program in one person with secondary progressive multiple sclerosis. The main findings of our study indicate that the intervention was well tolerated, feasible across both supervised clinical and home-based settings, and associated with improvements in functional mobility and spatiotemporal gait parameters over 18 weeks. Initially, the participant exhibited impairments affecting several components of gait, including reduced foot clearance during swing, impaired propulsion during late stance, shortened step length, balance deficits, and fatigue-related gait deterioration. Consequently, a multichannel FES system was considered particularly relevant, as it enabled simultaneous assistance of both distal muscles involved in foot clearance (tibialis anterior and fibularis longus) and proximal muscle groups contributing to weight acceptance, limb advancement, postural stability, and propulsion (quadriceps, hamstrings, gluteals, and triceps surae).

The primary objective of this case report was to assess feasibility, tolerance, and safety, which are critical considerations in PwMS given the high prevalence of fatigue, sensory disturbances, and fluctuating symptoms. The participant completed all planned sessions without dropout, demonstrating good adherence to both clinic-based and home-based phases. This finding is consistent with previous findings in incomplete SCI (9, 11), chronic neurological disorders (11), and post-stroke people (8). In addition, walking speed on the 10-m Walk Test increased by 58%–96% and TUG time decreased by 35%–38% across the intervention. These changes were compared against published MS-specific MDC values: 1.3 s for the TUG (23), 20%–36% for walking speed (24), and 8.28 cm for the FRT (25). TUG and 10-m Walk Test changes exceeded measurement error; the FRT gain approximated the MDC at MID1, exceeded it at MID2, and returned to no change at POST, thresholds derived in populations with generally lower EDSS than this participant. Improvements in the Functional Reach Test further suggest enhanced potential dynamic balance and postural control, which are critical determinants of fall risk in PwMS. These results are consistent with the existing literature, where studies reported significant improvements in walking speed in PwMS using FES (3, 26, 27). A more granular analysis of the session-by-session data (Figure 2) showed higher gait speeds and longer step lengths during the supervised phases compared with the unsupervised home-based phase. During the final phase, gait performance appeared more stable, with less variability between sessions. However, given the single-case design, the reasons underlying these differences remain speculative and may reflect the combined influence of supervision, motivation, day-to-day variability, fatigue, and training progression. The reasons for this dissociation remain unknown and warrant dedicated investigation in future studies.

The clinic-to-home rehabilitation design represents a notable strength of this case report. While improvements were already evident at MID (after the clinic-based phase), further gains were observed during the home-based phase, suggesting that continued training outside the clinic may consolidate and extend functional benefits. This is consistent with the possibility that structured multimodal home-based training, of which FES was one component, can be maintained beyond direct supervision without evident safety concerns in this participant.

Several limitations must be acknowledged. First, this is a single-case retrospective analysis, which precludes causal inference and generalization. Second, no follow-up assessment was conducted to assess retention of gains after cessation of the intervention. Nevertheless, since the completion of the program reported in this case study, the participant has continued home-based FES sessions, which may have contributed to maintaining functional engagement, although this was not formally evaluated. Thus, future studies should include larger cohorts, controlled designs, and longer follow-up periods to assess the durability of effects. Third, family history and psychosocial context were not available in the retrospective clinical record. Despite these limitations, this case report provides preliminary evidence that a hybrid clinic-to-home, multichannel FES rehabilitation program is feasible and well-tolerated, with functional changes observed in this participant across both clinical and home-based settings. These findings support further, controlled investigation of multichannel FES as a rehabilitation strategy for PwMS.

## Patient perspective

Documented in the clinical record, the participant reported a perceived improvement in her walking performance, noting that she was able to cover longer distances in her neighbourhood even without wearing the FES device. The participant also reported that her performance was better when another person, particularly the physiotherapist, was present to motivate her.

## Data Availability

The original contributions presented in the study are included in the article/Supplementary Material, further inquiries can be directed to the corresponding author.
